# Supplementary material for: Optimal minimal residual disease threshold in pediatric acute myeloid leukemia: A retrospective cohort study based on the TARGET database
Source: PLoS Med. 2026 May 8;23(5):e1005088. doi: 10.1371/journal.pmed.1005088 (PMC13155632; doi:10.1371/journal.pmed.1005088)
Supplement: S1 Code — (ZIP) [file pmed.1005088.s002.zip › S2 code/PROJ8_4_tbl1/PROJ8_4_tbl1_seg2_0.pdf]

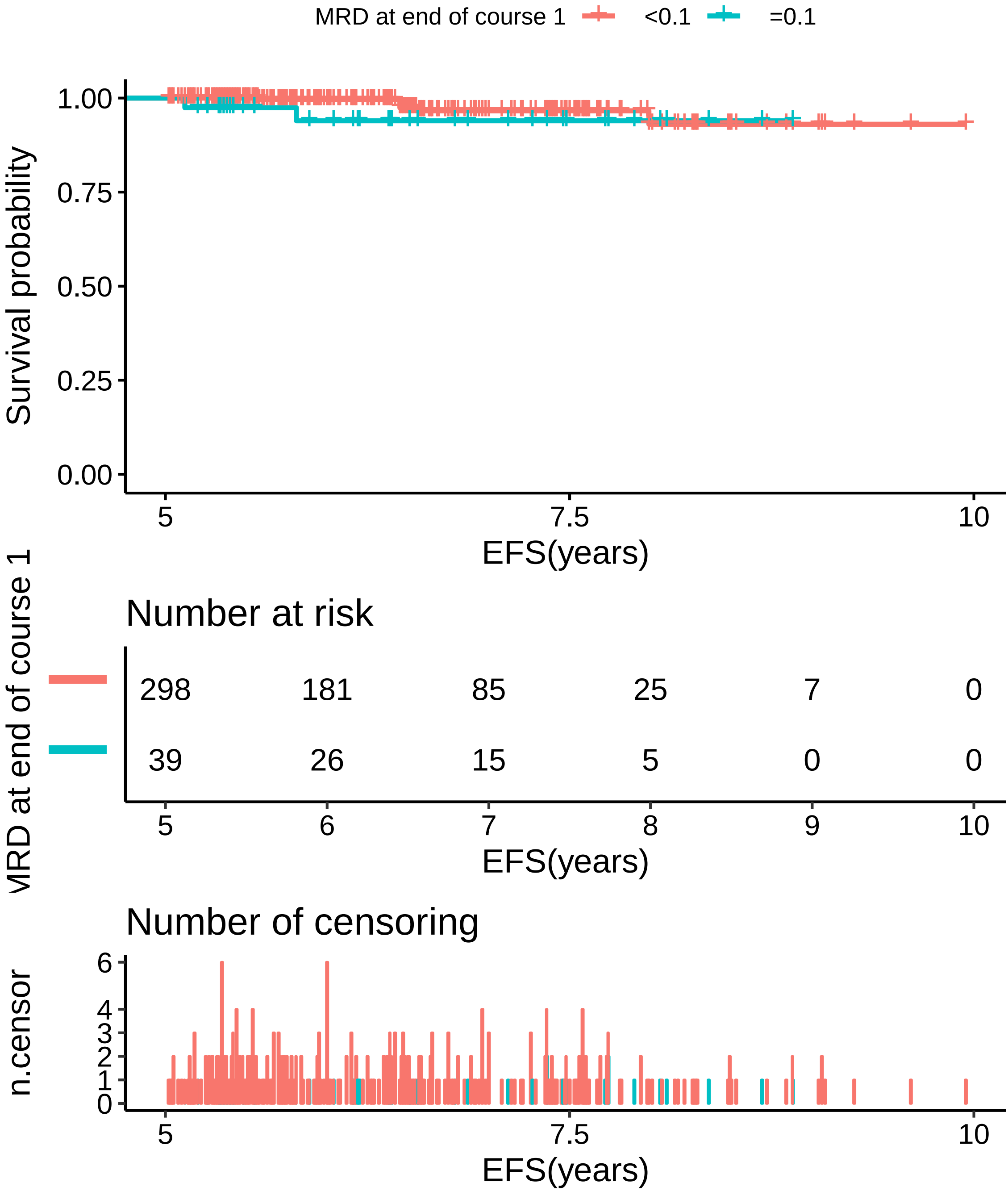
MRD at end of course 1
<0.1
=0.1
Survival probability
1.00
0.75
0.50
0.25
0.00
5
7.5
10
EFS(years)
Number at risk
MRD at end of course 1
298 181 85 25 7 0
39 26 15 5 0 0
5 6 7 8 9 10
EFS(years)
Number of censoring
n.censor
6
4
3
2
1
0
5
7.5
10
EFS(years)
